# Social Inequities in Exposure to Traffic-Related Air and Noise Pollution at Public Schools in Texas

**DOI:** 10.3390/ijerph20075308

**Published:** 2023-03-29

**Authors:** Jayajit Chakraborty, Jacob J. Aun

**Affiliations:** Department of Sociology & Anthropology, University of Texas at El Paso, El Paso, TX 79968, USA

**Keywords:** children, environmental justice, air pollution, road noise, public schools

## Abstract

Although children are particularly vulnerable to the adverse impacts of vehicular pollution and spend significant portions of their time at school, previous studies have not examined or compared school-level social inequities in exposure to both traffic-related air and noise pollution in the same study area. We addressed this gap through a case study in Texas—the second-largest US state based on total population and number of children. Vehicular pollution exposure was measured using: (1) outdoor concentrations of nitrogen dioxide (NO_2_), a widely used proxy for traffic-related air pollution; and (2) road noise estimates from the US Department of Transportation’s National Transportation Noise Mapping Tool. These variables were linked to data on locations and sociodemographic characteristics of children enrolled in Texas public schools. We found children attending schools with the highest exposure to both NO_2_ and road noise (top 25%) were significantly more likely to be Black, Hispanic, and eligible for free/reduced lunches (socioeconomically deprived). Results from multivariable generalized estimating equations that control for spatial clustering and other relevant factors revealed that schools with greater NO_2_ exposure were significantly more likely to serve racial/ethnic minority and younger students, while schools with greater exposure to road noise were significantly more likely to serve socioeconomically deprived and older students. These findings underscore the urgent need to reduce both air pollution and noise exposure at school locations, especially in schools attended by higher proportions of socially disadvantaged children that are often additionally burdened with other challenges.

## 1. Introduction

Exposure to both air and noise pollution from transportation sources has been widely recognized to be harmful to human health and wellbeing. Because of their unique biological vulnerabilities and mobility patterns, children are more susceptible to the adverse effects of environmental pollution exposure than adults, which potentially affects their health and ongoing development into adulthood [1,2,3,4]. Although their residential locations are relatively dispersed, children in the US are confined to school locations for an average of 6.64 h a day and more than 1200 h every year [5]. Schools are often located near heavily trafficked roadways that generate excessively high levels of vehicular air and noise pollution [6,7,8,9]. Children’s exposure to both air pollutants and excessive noise has been linked to lower student test scores, lower grade point averages, lower attention retention, and chronic absenteeism [10,11,12,13,14,15], as well as negative health outcomes such as respiratory illness, reduced lung function, diminished cognitive function, and mental health problems [16,17,18,19,20,21,22,23,24].

An emerging body of research has applied a distributive environmental justice framework to examine spatial and statistical associations between transportation-related pollution risks and sociodemographic characteristics of enrolled children in schools. Studies focusing on traffic proximity have found that schools with higher proportions of racial/ethnic minority and socioeconomically disadvantaged students locate near major highways and roadways with elevated traffic volumes [7,25,26,27,28]. Studies focusing on traffic-related air pollutants [29,30,31] and excessive road noise [8] also indicate that minority and socioeconomically disadvantaged children are exposed to significantly greater pollution levels than White and socioeconomically advantaged children at school locations. These findings collectively point to environmental inequities that require more detailed investigation using a comparative research approach which allows parallel analyses of both outdoor concentrations of air pollutants and excessive noise from transportation sources. While social disparities associated with exposure to air and noise pollution for school children have been examined separately, previous studies have not attempted to analyze inequities in exposure to both air and noise pollution in the same study area and thus provide a more comprehensive understanding of the socio-spatial implications of transportation-related health hazards in a particular place.

Texas is a particularly appropriate state for studying children’s exposure to vehicular pollution since it ranks second in terms of total number of children (i.e., population under 18 years) and the first in number of children below poverty level among all US states [32]. Media reports also suggest that many schools and school districts in Texas suffer from exceedingly high levels of transportation-related air and noise pollution [33]. A recent study found greater proportions of racial/ethnic minority and socioeconomically vulnerable children to live within school districts exposed to significantly higher levels of traffic proximity and related air pollution emissions [4]. To our knowledge, no previous study has analyzed exposure to vehicular air or noise pollution and related disparities for school children statewide in Texas.

This paper aims to address previous gaps and extend research on environmental injustices imposed on children by analyzing exposure inequities associated with both air pollution from transportation sources and excessive noise from roadways, at public schools in Texas. Our study seeks to answer two research questions: 

1. Are schools that are disproportionately exposed to higher levels of traffic-related air and noise pollution characterized by significantly greater percentages of racial/ethnic minority and socioeconomically deprived students? 

2. How do the racial/ethnic and socioeconomic characteristics of schools, as well as the grade level of students served by schools, relate to levels of traffic-related air and noise pollution exposure, after accounting for spatial clustering and other relevant factors? 

We measured exposure to traffic-related air pollution at the school level using average annual concentrations of nitrogen dioxide (NO_2_), a key contributor and widely used proxy for vehicular air pollution. Exposure to roadway noise is measured using data from the US Department of Transportation (USDOT)’s National Transportation Noise Mapping Tool. Statistical analyses are based on bivariate comparisons and multivariable generalized estimating equations that account for geographic clustering of public schools within school districts in Texas.

## 2. Materials and Methods

Public schools in Texas represent the unit of analysis for this study. The geographic locations of 8428 schools for the 2016–2017 school year were downloaded from the Texas Education Agency (TEA) Public Open Data portal [34] as a geographic information system (GIS) map layer. Data on school enrollment characteristics (2016–2017) were obtained from the National Center for Education Statistics, using the ELSi Table Generator tool [5].

### 2.1. Dependent Variables

The first dependent variable is average annual concentrations of NO_2_, a well-documented indicator of vehicular air pollution [35,36,37] that has been utilized in recent research on environmental injustices for children [4,31]. We used estimates of outdoor NO_2_ concentrations developed by the Center for Air, Climate and Energy Solutions (CACES), as described in Kim et al. [38]. Their empirical models are based on concentration measurements from US Environmental Protection Agency (EPA) regulator monitors, and combine land-use regression and satellite-derived estimates to predict concentrations for criteria air pollutants. For this study, census block-group-level values of predicted NO_2_ spatial concentrations and locational coordinates of block-group centroids were downloaded from the CACES Land Use Regression database for 2011 to 2015 (latest year available). Mean annual concentrations for this five-year timeframe (2011–2015) for all block groups in Texas were estimated and used, as recommended by recent studies utilizing this data source [6,39]. For each Texas public school, NO_2_ exposure was then calculated using values from the block group in which the school’s geographic coordinates were located.

Our second dependent variable, exposure to roadway transportation noise, was based on data from the National Transportation Noise Mapping Tool (NTNMT), developed by the USDOT’s Bureau of Transportation Statistics, which has also been utilized in recent noise-related environmental justice studies [8,40]. Data on road noise levels in the NTNMT in 2016 (earliest year available) for Texas were extracted and downloaded as a GIS raster map layer, available at a 30 m by 30 m pixel resolution. Noise measures are generated using a 24 h equivalent sound level (LAeq,24) metric and measured in A-weighted decibels (dB(A)), representing estimated average noise energy from transportation noise sources over a 24 h period at specified receptor locations. The NTNMT assumes that 24 h roadway traffic and aircraft-related noise levels below 45 dB(A) are not harmful. Noise measures are thus estimated in the NTNMT for Texas locations with modeled LAeq,24 values greater than or equal to 45 dB(A) from road transportation sources; zero values are assigned for other locations. More details on data sources and methodology employed by the NTNMT to develop road noise estimates for 2016 are available in USDOT [41]. 

The dependent variable representing exposure to road noise was estimated using a 500 m circular buffer around each public school, following previous studies on noise exposure [8,40,42]. We calculated the average noise level (LAeq,24) in dB(A) for all 30 m by 30 m pixels from the NTNMT whose centroids were located within a 500 m radius of each Texas public school, and used this measure as the dependent variable for road noise exposure.

Descriptive statistics for our dependent variables are provided in Table 1. Although no public school (0%) in Texas experienced NO_2_ exposure that exceeded the EPA’s annual average standard of 53 ppb, road noise levels were higher than the USDOT’s definition of harmful noise (45 dB(A)) in 7810 or 92.7% of all public schools in Texas.

### 2.2. Independent Variables

For each school, we obtained the number of students who were White, Black or African-American, and Hispanic. Asian and Pacific Islander students were combined into a single Asian/Pacific Islander category due to their smaller counts. Students who were of more than one race or belonged to other non-White races were also combined to comprise the multiracial/other-race category. Following previous studies on exposure disparities at school locations [8,12,30,31], we used students who qualify for free or reduced lunches as a proxy for socioeconomic deprivation in schools, since student families must be at or below 185% of the federal poverty line to become eligible. 

For the second research question, which involves multivariable modeling, the total number of enrolled children was used to estimate respective percentages for these racial/ethnic and socioeconomic categories; the White student percentage was excluded as the reference group. Our analysis for this research question also included dichotomous variables that represent the grade level of students served in each school using data on the highest grade offered at the school. These binary indicators comprise schools that serve children at or below the first grade (pre-elementary) and schools with the highest grade being second through sixth (elementary). We excluded schools serving seventh or higher grades as their highest grade (i.e., junior-high and high schools) from our models as the reference group, since older children are less vulnerable to the harmful effects of air and noise pollution than younger children [1,14,30]. To account for urban–rural differences in school location, we used Rural–Urban Commuting Area (RUCA) codes developed by the US Department of Agriculture [43]. Our models included dichotomous variables to identify schools located in metropolitan (RUCA codes 1–3) and micropolitan (RUCA codes 4–6) census tracts. Schools from tracts located in small towns and rural areas (RUCA codes 7–10) served as the reference group. School size, or the total number of enrolled students, was also used as an additional control variable, following studies that have previously examined air pollution [30] or noise exposure [8] at the school level. 

The names and summary statistics for independent variables used in this study are provided in Table 1.

### 2.3. Statistical Analysis

For the first research question, we first identified public schools located in the four quartiles for both NO_2_ and road noise exposure. We then estimated the percentages of students attending public schools in Texas belonging to each racial/ethnic category and eligible for free/reduced-price lunches, in each quartile. A z-test for difference in proportions was implemented to determine if the percentages of racial/ethnic minorities and socioeconomically deprived students in the highest quartile (top 25%) significantly exceeded those in other Texas schools (bottom 75%) and the first quartile (bottom 25%), separately for NO_2_ and noise exposure. These children-level bivariate comparisons included 5,340,966 students attending all 8428 Texas public schools with at least one enrolled student and no missing data.

For the second research question, we employed generalized estimating equations (GEEs), a multivariable statistical technique that extends the generalized linear model to accommodate clustered data, to predict the two dependent variables representing NO_2_ and road noise exposure, respectively. To ensure stable percentages for all independent variables, 176 schools with fewer than 30 children were excluded. Our multivariable analysis thus encompasses 8252 schools with no missing data for our variables. GEEs are suitable here because our data are clustered (e.g., schools within school districts) and because they relax several assumptions of traditional regression (e.g., normality). These models assume that observations from different clusters are not related to each other, while observations within a cluster (e.g., schools belonging to the same school district) are related. For each dependent variable and GEE, we selected model specifications that provided the best statistical fit based on the quasi-likelihood under the independence model criterion. Both models control for clustering utilizing the school district where the school was located (8252 schools in 1202 districts). All continuous independent variables were standardized before inclusion in the GEEs, and two-tailed p-values from the Wald Chi-square test were used to test the statistical significance of each variable coefficient. Diagnostic testing, based on variance inflation factor, tolerance, and condition index criteria, confirmed that the models were not affected by multicollinearity.

## 3. Results

**Figure 1:**
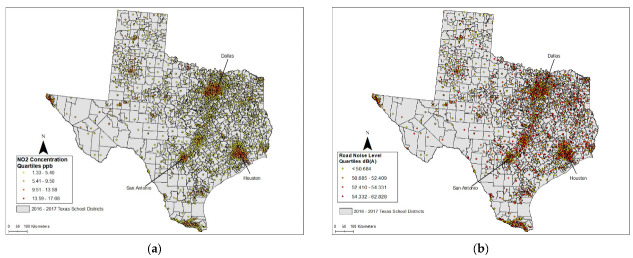
(**a**) School-level distribution of average NO_2_ concentration in parts per billion (ppb): 2011–2015; (**b**) school-level distribution of road noise level in A-weighted decibels (dB(A)): 2016.

The spatial distributions of the two dependent variables are shown in Figure 1, where all Texas public schools are grouped into four quartiles based on their respective values of NO_2_ concentrations (Figure 1a) and road noise (Figure 1b). Tracts in the highest quartile (top 25%) for both NO_2_ and noise exposure are located mainly within or adjacent to the three largest metropolitan areas (Dallas, Houston, and San Antonio) and along interstate highways that connect these areas. In contrast, schools in the lowest quartile (bottom 25%) for both dependent variables can be observed mainly in rural districts with fewer schools. However, a large proportion of schools in the highest quartile of road noise exposure are also located along secondary highways that intersect smaller metropolitan areas (e.g., Austin, Beaumont, and Lubbock) of Texas.

For the first research question, we compared the characteristics of enrolled students across the four quartiles depicted in Figure 1. These results are presented in Table 2. Students attending schools in the highest quartile (Q4) for NO_2_ concentration levels are significantly more likely to be Hispanic, Black, or Asian/Pacific Islander, and be eligible for free/reduced lunches, when compared to other schools in Texas and the lowest quartile (Q1), in particular. The largest disparities with respect to highest quartile were observed for Hispanic students and those eligible for free/reduced lunches. However, students attending schools in the highest quartile (Q4) for NO_2_ exposure are significantly less likely to be White or multi-racial/other race. The percentage of White students is highest and substantially larger in the lowest quartile (Q1) for NO_2_ exposure, compared to other quartiles. We found similar disparities for road noise exposure, in terms of significantly greater percentages of Black, Hispanic, and socioeconomically deprived students attending schools in the highest quartile (Q4) than other schools or the first quartile. However, these percentage differences were considerably smaller compared to those observed earlier for NO_2_ exposure. Additionally, students attending schools in the highest quartile (Q4) for road noise exposure are significantly less likely to be Asian/Pacific Islander or multi-racial/other race. The percentage of White students is again highest in the lowest quartile (Q1) for road noise exposure, but their percentages in the highest quartile (Q4) do not differ significantly from those in other Texas schools.

For the second research question, we used multivariable GEE models. Results from the GEE model for NO_2_ exposure are summarized in Table 3. The numbers in the Exp(Beta) column represent the percentage change in NO_2_ concentration for every one standard deviation increase in each of the independent variables, and can be used to interpret the observed statistical associations.

After controlling for clustering by school district (boundaries depicted in Figure 1) and other independent variables, the percentages of Hispanic, Black, Asian/Pacific Islander, and multi-racial/other-race children indicated a statistically significant and positive relationship with NO_2_ exposure (*p* < 0.001). Specifically, a one standard deviation increase in the percentage of Hispanic, Black, Asian/Pacific Islander, and multi-racial/other-race students is associated with 35.9%, 17.7%, 12.1%, and 4.9% increases in NO_2_ concentration levels, respectively. The relationship with socioeconomic deprivation (free/reduced-price lunches), however, was negative (*p* < 0.001). Both pre-elementary and elementary schools as well as those located in metropolitan tracts also showed significantly greater NO_2_ exposure (*p* < 0.05).

Multivariable GEE results for excessive road noise exposure are shown in Table 4. After adjusting for clustering by school district and other independent variables, the percentages of Hispanic, Black, Asian/Pacific Islander, and multi-racial/other-race children did not indicate a statistically significant association with road noise exposure (*p* > 0.05). However, the relationship with socioeconomic deprivation was positive and significant (*p* < 0.001). Specifically, a one standard deviation increase in the percentage of students eligible for free/reduced lunches is associated with a 3.8% increase in road noise levels. Both pre-elementary and elementary schools showed significantly lower noise levels compared to higher grade schools, although the association with pre-elementary was non-significant (*p* > 0.05). Schools in metropolitan tracts also indicated significantly greater road noise exposure (*p* < 0.05).

## 4. Discussion

This study focused on investigating racial/ethnic and socioeconomic inequities in exposure to both traffic-related air and noise pollution across public schools in Texas. With regard to our first question, schools exposed to the highest levels of both outdoor NO_2_ and road noise (top 25%) were found to contain a significantly greater proportion of enrolled students who are Black and Hispanic, with the Hispanic percentage showing the highest deviations or greatest disparity, compared to other Texas schools (bottom 75%). In contrast, the White student percentage was consistently higher in schools facing the lowest exposure (bottom 25%). Socioeconomically deprived students were also significantly overrepresented in schools with highest exposure (top 25%) to both NO_2_ and road noise.

Our second research question focused on analyzing exposure inequities based on sociodemographic characteristics and grade level of schools, after adjusting for school size, urban/rural location, and spatial clustering of schools by school district. Multivariable models revealed significantly higher percentages of Hispanic, Black, and Asian/Pacific Islander to be associated with greater NO_2_ exposure. The strongest positive relationship with outdoor NO_2_ concentration levels was observed for the Hispanic student percentage, followed by the Black student percentage. While similar racial/ethnic disparities were not observed for road noise exposure, a higher percentage of socioeconomically deprived students was found to be significantly related to greater noise exposure levels. The non-significant associations with Hispanic and Black percentages in our multivariable analysis can be explained, in part, by higher road noise levels around schools in smaller metropolitan areas (e.g., Abilene, Amarillo, Lubbock, and Midland) that are characterized by relatively higher proportions of White students compared to the more racially/ethnically diverse schools in the largest metropolitan areas (e.g., Dallas, Houston, and San Antonio) of Texas. While public schools in these large urban areas may not be exposed to excessive traffic-related noise, they could experience higher noise levels from various non-transportation sources (e.g., construction and industrial).

Our results for the relationship between NO_2_ exposure and the proportions of Hispanic, Black, and Asian/Pacific Islander school children in Texas are consistent with those from previous studies that reported racial/ethnic disparities in exposure to ambient air pollution at school locations [26,27,28,29,30,31,44]. While limited environmental justice research on road noise exposure has been conducted in the US, our results for socioeconomically deprived students are similar to previous studies that found greater noise levels in schools with higher proportions of economically deprived students [8] or neighborhoods of lower socioeconomic status [40,45,46]. These findings emphasize the need for additional empirical research on exposure to vehicular air and noise pollution for socially disadvantaged school children in other US cities and states, as well as in other countries of the world.

In terms of other explanatory factors, we found divergent exposure patterns for NO_2_ and roadway noise. Multivariate model results revealed that younger children (i.e., elementary school or earlier grade levels) are exposed to significantly higher outdoor NO_2_ concentrations than older children (i.e., junior-high or high school levels) while attending public schools in Texas. These results are a cause for serious concern, since younger children are more vulnerable to the effects of toxic air pollutants because of heavier exposures (i.e., consuming more air and food per unit of body weight compared to older children and adults), biologic sensitivity linked to their ongoing growth and development, and their long future lifetimes since early insults can adversely impact their health and wellbeing as adults [1,14,30,47]. With regard to road noise levels, however, elementary school children indicated significantly lower exposure compared to older children who attend junior-high or high schools. Although these results do not align with those reported by the only national-scale environmental justice study of public schools in the US [8], another noise-related study found children to be exposed to lower noise levels than individuals aged more than 18 years [40].

While our study provides new insights on social inequities in traffic-related air pollution and noise exposure across Texas public schools, it is important to consider three limitations. First, our analysis of air pollution exposure at school locations is based on modeled estimates of ambient, annual average NO_2_ concentrations. Ambient concentrations of pollutants could differ from personal exposure estimates, especially when children do not spend all of their time at schools and may also spend more time indoors than outdoors while at school [31]. Consequently, our results represent surrounding outdoor air as a proxy for exposure during school hours. Second, since children are only present at schools during the day, it would have been more appropriate to examine average daytime instead of 24 h road noise measures. Although previous studies have used 24 h average noise levels for analyzing exposure disparities for schools, these metrics potentially underestimate daytime traffic-related noise at school locations [8], especially during hours when traffic volume is highest (i.e., rush hour). Third, the percentage of students eligible for free or subsidized lunches may not serve as the most reliable indicator for poverty and should not be confused with direct measures of socioeconomic status that require more detailed information on financial factors or household income for each enrolled student [31].

## 5. Conclusions

This paper contributes to the growing body of research on environmental injustices experienced by children by presenting the first quantitative analysis of exposure to traffic-related air and noise pollution at public schools in Texas—the second largest and one of the most environmentally polluted US states. Unlike previous studies on schools that focus either on air pollutants or noise levels, we utilized variables that encompass both these dimensions and are based on the most geographically detailed estimates of air pollution and noise exposure from transportation sources currently available. Our findings reveal specific differences in the socio-spatial patterns of traffic-related air pollution and road noise exposure across public schools in Texas, thus emphasizing the need to examine inequities associated with both these environmental hazards. Schools burdened by greater NO_2_ exposure are more likely to serve racial/ethnic minority and younger children (i.e., elementary or lower levels) and locate in both metropolitan and micropolitan areas of the state. In contrast, schools experiencing greater exposure to road noise are more likely to serve socioeconomically deprived and older children (i.e., junior-high or high school levels) in metropolitan areas of Texas.

These racial/ethnic and socioeconomic inequities associated with traffic-related air and noise pollution have important policy implications, because children’s exposure to both these hazards have been associated with adverse health outcomes that cause school absence, poorer academic performance, increased probabilities of suboptimal health and diminished achievement as adults, and perpetuation of intergenerational disenfranchisement and poverty [2,3,4,21,48]. Our findings thus underscore the urgent necessity to develop and implement appropriate mitigation strategies that focus on reducing school exposure to both vehicular air and noise pollution, especially in schools attended by higher percentages of racial/ethnic minority and socioeconomically deprived children that are often additionally burdened with other challenges such as limited financial resources, smaller annual budgets, and teacher shortages that negatively impact their students.

## Figures and Tables

**Table 1:** Summary statistics for variables analyzed.

	Min.	Max.	Mean	SD
Dependent variables:				
*Air pollution exposure*: mean NO_2_ concentration in parts per billion in census block group of school location: 2011–2015	1.327	17.676	6.338	2.664
*Noise exposure*: road noise level in A-weighted decibels (dB(A)) within 500 meters of school: 2016	0.000	62.828	48.919	13.977
Independent variables:				
Total number of students	1	5947	634	523
% Students: White	0.000	100.000	50.930	29.687
% Students: Hispanic or Latino	0.000	100.000	50.930	29.687
% Students: Black or African-American	0.000	100.000	12.085	16.132
% Students: Asian or Pacific Islander	0.000	84.570	3.110	7.042
% Students: multi-racial or other minority race	0.000	33.333	2.688	2.384
% Students: free or reduced lunch eligible	0.000	99.849	55.262	26.708
Highest grade served: pre-elementary (early education–1st)	0.000	1.000	0.003	n/a
Highest grade served: elementary (2nd–6th)	0.000	1.000	0.530	n/a
Metropolitan (RUCA code of census tract: 1–3)	0.000	1.000	0.800	n/a
Micropolitan (RUCA code of census tract: 4–6)	0.000	1.000	0.090	n/a

**Table 2:** Distribution of Texas public school students across traffic-related air and noise pollution exposure quartiles.

	Q1: Lowest 25%	Q2	Q3	Q4: Highest 25%	Q4-Other Schools ^1^	Q4–Q1: Difference
*NO_2_ concentration in parts per billion:*						
% Students: White	49.36%	34.06%	22.92%	14.71%	−18.49% **	−34.64% **
% Students: Hispanic or Latino	39.46%	47.63%	52.42%	65.11%	17.49% **	25.65% **
% Students: Black or African-American	6.75%	11.27%	16.00%	13.74%	1.63% **	6.99% **
% Students: Asian or Pacific Islander	1.46%	4.02%	5.85%	4.36%	0.21% **	2.90% **
% Students: multi-racial or other minority	2.97%	3.02%	2.82%	2.08%	−0.85% **	−0.89% **
% Students: free or reduced lunch eligible	46.74%	45.66%	51.65%	65.06%	16.70% **	18.32% **
*Road noise level in dB(A):*						
% Students: White	30.45%	26.25%	27.89%	28.13%	−0.01%	−2.32% **
% Students: Hispanic or Latino	50.41%	52.06%	53.03%	54.13%	3.50% **	3.07% **
% Students: Black or African-American	12.11%	11.02%	11.98%	15.18%	2.30% **	3.72% **
% Students: Asian or Pacific Islander	4.18%	4.78%	4.45%	3.39%	−1.09% **	−0.79% **
% Students: multi-racial or other minority	2.85%	2.71%	2.65%	2.56%	−0.18% **	−0.28% **
% Students: free or reduced lunch eligible	51.16%	53.00%	53.82%	53.77%	1.11% **	2.61% **

Note: ** *p* < 0.01, based on two-sample z-test of proportions; ^1^ Other schools represent public schools in the lowest 75% for NO_2_ exposure and road noise level, respectively.

**Table 3:** Multivariable generalized estimating equation (GEE) for predicting NO_2_ exposure in Texas public schools.

	Beta	Lower 95% CI	Upper 95% CI	Exp(Beta)	*p*-Value
Total number of students	0.006	−0.005	0.017	1.006	0.294
% Students: Hispanic or Latino	0.307	0.279	0.336	1.359	<0.001
% Students: Black or African-American	0.163	0.135	0.192	1.177	<0.001
% Students: Asian or Pacific Islander	0.114	0.091	0.137	1.121	<0.001
% Students: multi-racial/other minority	0.048	0.026	0.069	1.049	<0.001
% Students: free/reduced lunch eligible	−0.065	−0.094	−0.036	0.937	<0.001
Pre-elementary school	0.053	0.012	0.094	1.054	0.011
Elementary school	0.022	0.002	0.042	1.022	0.030
Metropolitan	0.316	0.267	0.366	1.372	<0.001
Micropolitan	0.017	−0.034	0.069	1.017	0.509
Intercept	1.535	1.499	1.572		<0.001
Scale	0.017				

Note: GEE is based on an inverse Gaussian distribution with a logarithmic link function and an independent correlation matrix; N = 8252 schools with at least 30 enrolled students.

**Table 4:** Multivariable GEE for predicting road noise exposure in Texas public schools.

	Beta	Lower 95% CI	Upper 95% CI	Exp(Beta)	*p*-Value
Total number of students	0.001	−0.005	0.008	1.001	0.719
% Students: Hispanic or Latino	−0.015	−0.032	0.002	0.985	0.088
% Students: Black or African-American	−0.002	−0.013	0.009	0.998	0.757
% Students: Asian or Pacific Islander	0.005	−0.006	0.015	1.005	0.364
% Students: multi-racial/other minority	−0.007	−0.018	0.005	0.993	0.259
% Students: free/reduced lunch eligible	0.037	0.019	0.055	1.038	<0.001
Pre-elementary school	−0.012	−0.043	0.019	0.988	0.463
Elementary school	−0.039	−0.054	−0.025	0.962	<0.001
Metropolitan	0.033	0.003	0.064	1.034	0.031
Micropolitan	0.018	−0.016	0.053	1.018	0.301
Intercept	3.882	3.854	3.910		<0.001
Scale	2.136				

Note: GEE is based on a Tweedie distribution with a logarithmic link function and an independent correlation matrix; N = 8252 schools with at least 30 enrolled students.

## Data Availability

Data available on request from the authors.
